# Autoimmune Hepatitis (AIH) Related to Drug-Induced Liver Injury (DILI) Due to Atorvastatin

**DOI:** 10.7759/cureus.82315

**Published:** 2025-04-15

**Authors:** Jaime Loeza-Suárez, Diana Citlalli García-Luna, Janely Hernández-Mirón, Raúl Ramírez-Marcial, Grissell Alejandra Valero-Gaona

**Affiliations:** 1 Internal Medicine, Hospital Juárez de México, México City, MEX; 2 Internal Medicine, Hospital Juárez de México, Mexico City, MEX; 3 Gastroenterology and Hepatology, Hospital Juárez de México, Mexico City, MEX; 4 Rheumatology Department, Instituto Mexicano del Seguro Social (IMSS), México City, MEX

**Keywords:** atorvastatin-induced autoimmune hepatitis, autoimmune hepatitis (aih), drug-induced liver injury (dili), statin, statin-induced hepatitis

## Abstract

Drug-induced liver injury (DILI) is a rare condition characterized by hepatotoxic damage, identified through the elevation of liver function tests associated with a specific drug. Statins are among the medications linked to DILI, although this association is infrequent. Recently, reports and case series have described a relationship between atorvastatin-induced DILI and autoimmune hepatitis (AIH), two conditions not commonly associated. Because there are no established clinical or diagnostic criteria for this overlap, recognition is complex and relies on the clinical course, a timeline of causality, and the exclusion of alternative diagnoses. This report presents the case of a patient in the seventh decade of life who developed atorvastatin-induced DILI, meeting the diagnostic criteria for AIH. We discuss the therapeutic approach and review the relevant literature on this rare association.

## Introduction

Drug-induced liver injury (DILI) is a rare condition characterized by hepatotoxic injury, evidenced by elevated liver enzymes in association with a specific drug [1]. Statins are among the medications that may cause DILI; however, this association remains uncommon and, in some cases, controversial [2,3]. Autoimmune hepatitis (AIH) is a disease of unknown etiology, defined by elevated liver enzymes and the presence of characteristic autoantibodies [4]. Its association with drug exposure has been infrequently reported, and the diagnosis is based solely on a causal relationship between drug exposure and subsequent signs and symptoms [1].

This report describes a patient who met the Roussel Uclaf Causality Assessment Method (RUCAM) and Revised Electronic Causality Assessment Method (RECAM) criteria for the diagnosis of atorvastatin-induced DILI, along with biochemical, immunological, and histological criteria for AIH.

## Case presentation

A 65-year-old man with a history of type 2 diabetes had been hospitalized two months prior due to an acute myocardial infarction, which was managed with percutaneous coronary intervention and stenting in the right coronary artery. His treatment regimen included clopidogrel (75 mg), metoprolol (200 mg), empagliflozin (12.5 mg), and atorvastatin (80 mg) every 24 hours. The patient developed vomiting and epigastric pain radiating to the right colic frame. Subsequently, hiccups, Bristol 5 evacuations, and jaundice appeared. Laboratory tests were performed, and he was referred to our hospital. On admission, direct pattern hyperbilirubinemia, hyperkalemia, acute kidney injury with elevated nitrogen levels, and elevated aminotransferases were detected (Table 1).

**Table 1:** Laboratory studies Renal injury stands out and alteration of liver function tests.

	Value	Reference range
Calcium	8.2 mg/dL	8.4-10.4 mg/dL
Chlorine	100 meq/L	98-109 meq/L
Potassium	6.5 meq/L	3.5-5.5 meq/L
Sodium	133 meq/L	132-146 meq/L
Creatinine	12.07 mg/dL	0.5-1.3 mg/dL
Glucose	142 mg/dL	70-110 mg/dL
BUN	238 mg/dL	9-23 mg/dL
Urea	509 mg/dL	10-50 mg/dL
Direct bilirubin	3.09 mg/dL	0.01-0.30 mg/dL
Total bilirubin	3.86 mg/dL	0.01-1.0 mg/dL
Alkaline phosphatase	451 UI/L	44-147 UI/L
Aspartate aminotransferase	2,357 U/L	10-39 U/L
Alanine aminotransferase.	3115 U/L	10-49 U/L
Leukocytes	10,920 U/L	5,200-12,400 U/L
Hemoglobin	13.9 g/dL	12-18 gr/dL
Platelets	125,000 U/L	130,000-400,000 U/L
Neutrophils	9,120 U/L	1,900-8,000 U/L
Immunoglobulin A	646 mg/dL	85-385 mg/dL
Immunoglobulin E	329 UI/mL	0-100 UI/mL
Immunoglobulin G	1450 mg/dL	560-1760 mg/dL
Immunoglobulin M	91.5 mg/dL	45-250 mg/dL

**Figure 1:**
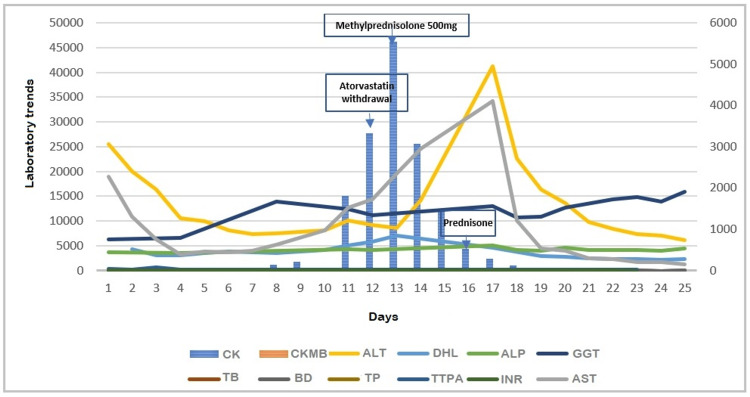
Laboratory trends Trends in laboratory parameters. CK: creatine kinase, CKMB: MB fraction of creatine kinase, ALT: alanine aminotransferase, DHL: lactate dehydrogenase, ALP: alkaline phosphatase, GGT: gamma-glutamyl transferase, TB: total bilirubin, BD: direct bilirubin, TP: thromboplastin time, APTT: activated partial thromboplastin time, INR: international normalized ratio, AST: aspartate aminotransferase. The timeline highlights the withdrawal of atorvastatin due to rhabdomyolysis, the administration of a methylprednisolone pulse, and the initiation of oral prednisone therapy.

Treatment with hyperhydration was initiated while maintaining the same medication regimen. However, early in hospitalization, creatine kinase levels increased, fulfilling the diagnostic criteria for rhabdomyolysis, leading to the discontinuation of atorvastatin (Figure 1). In addition, a decrease in muscle strength was observed in the pelvic limbs, more pronounced proximally than distally, raising suspicion of inflammatory myopathy. Autoantibody testing revealed positive anti-nuclear antibodies with a homogeneous nuclear pattern at a titer of 1:160 and mitochondrial cytoplasmic antibodies at a titer of 1:80 (Table 2). Antibodies associated with inflammatory myopathy were not detected. A muscle biopsy was performed, without finding data compatible with inflammatory myopathy.

**Table 2:** Serum antibodies Antibodies for inflammatory myopathy were tested due to muscle weakness and yielded negative results. Anti-smooth muscle antibody (ASMA) and anti-mitochondrial antibody tests were performed as part of the evaluation for liver disease, with positive results for ASMA and anti-nuclear antibody (ANA).

	Value	Reference range
Antinuclear antibodies	1:100 fine nucleolar pattern	<1:40
Anti-smooth muscle antibodies	1:80	C1:40
Anti-mitochondrial antibodies	1:40	<1:80
Anti ribosomal P antibodies	<20.0 U/mL	<20.0 U/mL
Anti-Jo-1 antibodies	<2.2 CU	2.2-20 CU

**Figure 2:**
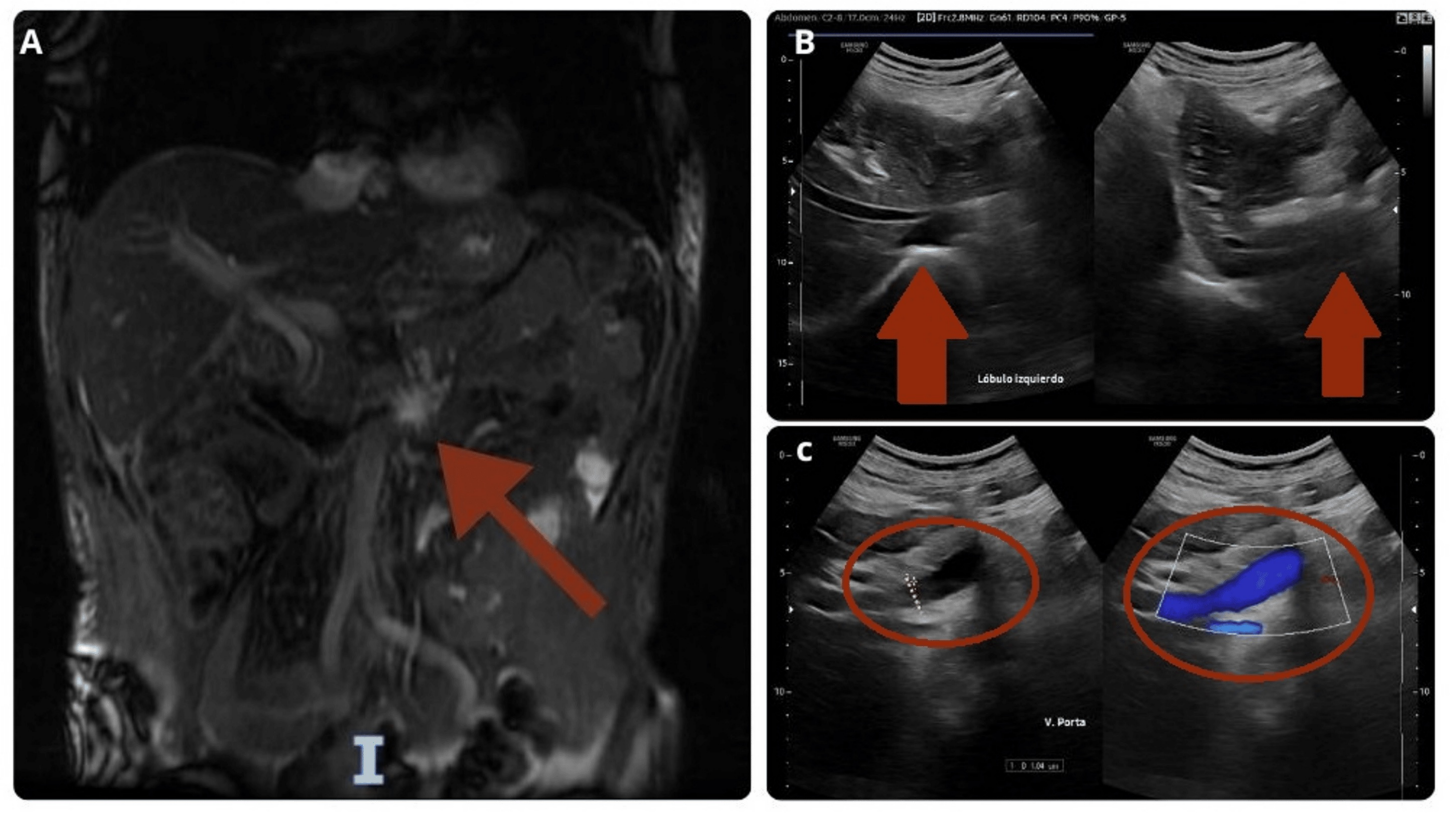
Liver ultrasound and magnetic resonance cholangiography A. Magnetic resonance cholangiography: The liver has a longitudinal diameter of 130 mm, with regular margins and no alterations in the intrahepatic biliary tract. The common bile duct measures 7 mm in diameter. B. Left lobe: A heterogeneous pattern is observed. C. Portal vein measures 10 mm. No anatomical abnormalities detected.

**Figure 3:**
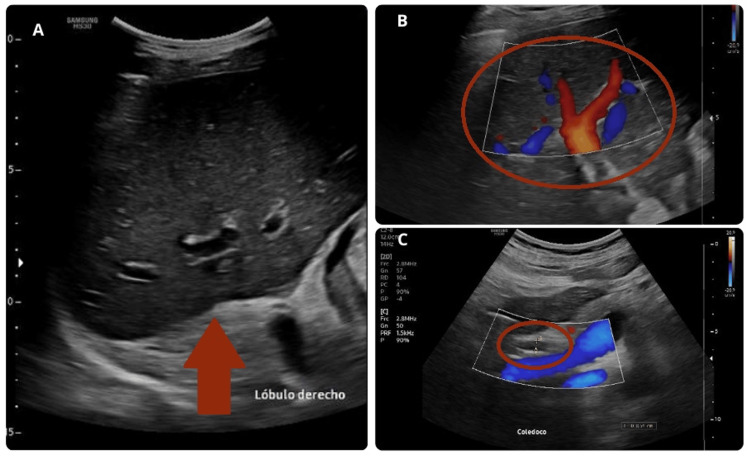
Liver ultrasound A. Right lobe. A heterogeneous pattern is observed by multiple echogenic images of diffuse distribution. B. Right lobe with color Doppler. C. Common bile duct measures 5 mm. No anatomical abnormalities detected, only changes in relation to the inflammatory process.

As part of the evaluation for liver injury, an ultrasound of the liver and bile ducts was performed, along with magnetic resonance cholangiopancreatography, both of which revealed no pathological findings (Figures 2, 3). Serological tests for hepatitis A, B, and C were negative. ELISA for HIV was performed, and the result was negative.

Because the patient presented with elevated aminotransferases, positive anti-nuclear antibodies, hyperbilirubinemia, and an R factor indicating a hepatocellular pattern (20.72 points), we performed antibody testing for AIH (Table 2). The results showed anti-smooth muscle antibody positivity. In the third week, a peak elevation in creatine kinase was observed (Figure 1). Administration of 500 mg of methylprednisolone resulted in substantial improvements in creatine kinase levels and muscle strength. The patient was discharged with a treatment regimen of azathioprine and prednisone in the context of AIH RUCAM and RECAM criteria were applied to atorvastatin exposure, yielding a total score of eight points (onset of symptoms five to 90 days after drug exposure: two points, reduction of alanine aminotransferase by more than 50% after drug withdrawal: two points, exclusion of other causes: two points, drug label evidence: two points), supporting a causal association consistent with atorvastatin-induced DILI [5].

After discharge, a liver biopsy was performed, revealing chronic hepatitis with a single focal limiting plate lesion and moderate stage 2 portal fibrosis, along with chronic cholestatic damage. No inflammatory infiltrate rich in plasma cells was identified; however, the lesion's topography, combined with chronic cholestatic damage, suggested AIH. No inflammatory damage or primary bile duct reduction was observed.

During outpatient follow-up, liver function tests and creatine kinase levels normalized. We decided to continue steroid therapy without interruption and avoid reintroducing atorvastatin.

## Discussion

Atorvastatin belongs to the statin class of drugs, exerting its mechanism of action through the inhibition of 3-hydroxy-3-methylglutaryl-coenzyme A reductase (HMG-CoA reductase), thereby blocking the initial steps of cholesterol metabolism [6]. Most of its metabolism occurs in the liver [6]. Over more than 20 years of clinical use, the most frequently reported adverse effects from atorvastatin have been gastrointestinal disorders; liver dysfunction is among the most concerning [7]. The most common finding is an asymptomatic and transient elevation of aminotransferases during the first weeks of treatment [8]. More severe manifestations include cholestasis and acute hepatitis [8].

DILI is characterized by liver injury detected through elevated liver enzymes in the blood, which may be associated with a specific drug [9]. It is classified as direct (or intrinsic) when caused by a hepatotoxic compound administered at standard doses, making it predictable and reproducible in animal models [9]. It is termed idiopathic when associated with commonly used medications at therapeutic doses, rendering it unpredictable and not reproducible [9]. Its incidence is approximately 15-20 cases per 100,000 individuals and contributes to half of all acute liver failure cases in Western countries [10].

Although atorvastatin has a known direct hepatotoxicity, its association with DILI remains rare, with only case reports and case series available in the literature. Diagnosis is challenging due to its low prevalence and the current lack of specific diagnostic tools [8]. Atorvastatin can be safely used in individuals with liver disease [2]. DILI due to statins is a rare and therefore unpredictable event [2]. Reports and documented case series have not necessarily linked its occurrence to prior liver disorders [2].

The RUCAM is the most widely used tool in DILI studies. The scoring system consists of eight sections divided into seven categories, with the final score interpreted as follows: 0 (excluded), 1-2 (unlikely), 3-5 (possible), 6-8 (probable), and >8 (highly probable) [5]. Due to technical limitations associated with the RUCAM, a revised electronic method, known as the RECAM, has recently been developed to improve objectivity. The RECAM has demonstrated superior performance in detecting extreme diagnostic categories of DILI [11]. In the present case, a score of 8 was obtained using both classifications, confirming a causal association between liver injury and atorvastatin administration and withdrawal.

Regarding AIH, its clinical presentation ranges from an insidious onset with fatigue, jaundice, and arthralgia to an acute and severe course with a poor prognosis [4]. The age distribution follows a bimodal pattern, with peaks occurring around puberty and between the fourth and sixth decades of life [4]. The characteristic biochemical profile includes hypergammaglobulinemia with selective IgG elevation, aminotransferase levels up to 50 times the upper limit, and, in some cases, elevated bilirubin [4]. Between 70% and 80% of patients with AIH have antinuclear antibody or anti-smooth muscle antibody titers of 1:40 or greater, whereas 3%-4% have anti-LKM1 antibodies, sometimes at trace levels below 1:40 [7]. In the present case, clinical, biochemical, and histological criteria consistent with AIH were met.

In the natural history of the disease, patients with AIH typically improve with immunosuppressive therapy; a rapid and sustained response to treatment supports the diagnosis [7]. In the present case, the patient’s symptoms improved following atorvastatin withdrawal and steroid therapy. After the diagnosis of AIH had been established, immunosuppressive treatment was continued; there was no subsequent disease activity, and liver function tests became normalized.

The association between DILI and AIH has been infrequently reported in the literature. Some authors consider these to be distinct entities with similar clinical manifestations, requiring differentiation rather than being considered a continuum of the same pathology [9]. Distinguishing between them presents a clinical challenge because no specific diagnostic criteria or tools currently exist to differentiate the conditions. Biomarkers such as high-mobility group box 1, keratin 18, and adenosine triphosphate have been proposed for DILI diagnosis. These markers represent damage-associated molecular patterns expressed when resident hepatic immune cells trigger an inflammatory response [9]. There is evidence that AIH may develop after drug exposure, initially presenting with a clinical course that resembles DILI [12].

Certain drugs have been associated with AIH phenotypes that persist after drug discontinuation, suggesting the activation of latent autoimmunity [13]. Currently, no consensus exists regarding the definitions, classifications, or criteria for immune-mediated DILI [12]. In 2011, Weiler-Normann and Schramm proposed a classification system to categorize patients with drug-mediated AIH into three groups: 1) AIH with DILI (patients with a known diagnosis of AIH, likely with a causal association, often presenting with advanced fibrosis on histology.); 2) drug-induced AIH (patients without a prior diagnosis of AIH or a known predisposition, in whom AIH develops following DILI; these patients respond well to steroids, achieve resolution after drug withdrawal, but may experience relapses after discontinuation of immunosuppression; the possibility of AIH as a first presentation cannot be excluded.); and 3) immune-mediated DILI (patients with clinical, biochemical, and histological features resembling AIH; eosinophilia and rash may be present, advanced fibrosis is generally absent, and the disease remains inactive after steroid withdrawal.) [12]. The present case met the criteria for classification as drug-induced AIH.

It is important to differentiate drugs as triggers of autoimmune liver disease from DILI [13]. A potential distinguishing factor is that immune-mediated DILI nearly always resolves after the withdrawal of both the causative drug and immunosuppressive therapy [13]. However, the assertion that relapse after steroid withdrawal distinguishes immune-mediated DILI from classic AIH is not entirely applicable to real-world clinical scenarios. In many cases, the coexistence of other autoimmune disorders prevents the discontinuation of treatment [13]. In the present case, steroid therapy has not been discontinued because it remains necessary for AIH treatment. Another approach that could help to clarify the diagnosis would involve re-administering atorvastatin. However, this would pose a substantial risk to the patient’s health and is therefore not recommended in this or most clinical contexts involving similar associations.

Case series and literature reviews indicate that statins may play a role in triggering or unmasking an autoimmune process; lupus and inflammatory myopathy are the most frequently associated conditions [14]. In the present case, atorvastatin was associated with both DILI and drug-induced AIH. Due to diagnostic uncertainty, a liver biopsy was performed, but the findings did not allow a clear distinction between these two conditions.

In a study by Suzuki et al., 63 liver biopsies from patients diagnosed with AIH (N=28) and DILI (N=35) were analyzed and compared. Inflammatory changes were observed in all cases of AIH and DILI, with more severe inflammation noted in AIH [15]. Portal and intra-acinar plasma cells, rosette formation, and emperipolesis were characteristic of AIH, whereas portal neutrophilia and hepatocellular cholestasis were more prevalent in DILI [15]. However, these findings are not pathognomonic because a considerable histological overlap was documented between AIH and DILI. Features commonly described as “typical” of AIH were also observed in a substantial proportion of DILI cases [15].

In summary, no clear distinguishing characteristics exist between immune-mediated DILI and AIH. Both conditions share clinical, histological, and serological features; a key difference is the tendency for relapse in AIH after discontinuation of immunosuppressive therapy [12]. It remains practically impossible to determine whether a patient who develops AIH after an episode of DILI was already predisposed to the disease or had pre-existing subclinical AIH before drug exposure.

## Conclusions

Atorvastatin-induced DILI is rarely reported in the literature. Although this drug has some hepatotoxic potential, it is also recognized as a trigger for autoimmune diseases. Reports and case series have described associations between DILI and drug-induced AIH. Both conditions exhibit overlapping clinical, biochemical, and histological characteristics. Few diagnostic tools are available to clearly differentiate between them, raising the suspicion that they represent variations of the same pathology. Further research is needed to establish clear distinctions between these conditions or determine whether they should be unified into a single nosological entity with defined clinical criteria, diagnostic standards, and therapeutic guidelines.
